# Validation of the 2020 AHA/ACC Risk Stratification for Sudden Cardiac Death in Chinese Patients With Hypertrophic Cardiomyopathy

**DOI:** 10.3389/fcvm.2021.691653

**Published:** 2021-08-17

**Authors:** Yan Dong, Wen Yang, Chongchong Chen, Jiamei Ji, Wei Zheng, Fengxiang Zhang, Bing Yang, Xiaorong Li, Xiujuan Zhou

**Affiliations:** ^1^Department of Cardiology, The First Affiliated Hospital of Nanjing Medical University, Nanjing, China; ^2^Department of Cardiology, Shanghai East Hospital, Tongji University School of Medicine, Shanghai, China

**Keywords:** hypertrophic cardiomyopathy, sudden cardiac death, risk stratification, guideline, Chinese patients

## Abstract

**Background:** Sudden cardiac death (SCD) is a common cause of death in hypertrophic cardiomyopathy (HCM), but identification of patients at a high risk of SCD is challenging. The study aimed to validate the three SCD risk stratifications recommended by the 2011 ACCF/AHA guideline, the 2014 ESC guideline, and the 2020 AHA/ACC guideline in Chinese HCM patients.

**Methods:** The study population consisted of a consecutive cohort of 511 patients with HCM without a history of SCD event. The endpoint was a composite of SCD or an equivalent event (appropriate implantable cardioverter defibrillator therapy or successful resuscitation after cardiac arrest).

**Results:** During a follow-up of 4.7 ± 1.7 years, 15 patients (2.9%) reached the SCD endpoint and 12 (2.3%) were protected by implantable cardioverter defibrillator for primary prevention. A total of 13 (2.8%) patients experiencing SCD events were misclassified as low-risk patients by the 2011 ACCF/AHA guideline, 12 (2.3%) by the 2014 ESC model, and 7 (1.6%) by the 2020 AHA/ACC guideline. The SCD risk stratification in the 2020 AHA/ACC guideline showed greater area under the curve (0.71; 95% CI 0.56–0.87, *p* < 0.001) than the one in the 2011 ACCF/AHA guideline (0.52; 95% CI 0.37–0.67, *p* = 0.76) and 2014 ESC guideline (0.68; 95% CI 0.54–0.81, *p* = 0.02).

**Conclusion:** The SCD risk stratification recommended by the 2020 AHA/ACC guideline showed a better discrimination than previous stratifications in Chinese patients with HCM. A larger multicenter, independent, and prospective study with long-term follow-up would be warranted to validate our result.

## Introduction

Hypertrophic cardiomyopathy (HCM) is one of the most common inherited cardiac diseases with a prevalence of 1/500 in the general population (1). Sudden cardiac death (SCD), heart failure, and thromboembolism are three main causes of death in HCM patients. Although the prevalence of SCD is relatively low with 0.5 to 1% per year (2, 3), SCD is a devastating clinical event once it happens. So it is critical to identify the high-risk patients to be protected from SCD by implantable cardioverter defibrillator (ICD).

The definitions of the SCD risk stratifications varied in the guidelines of the diagnosis and treatment of patients with HCM. The 2003 ACC/ESC guideline and the 2011 ACCF/AHA guideline showed limited power to distinguish high- from low-risk patients with lower specificity, leading to ICD implantation in a large number of patients who did not experience SCD events (4–6). The 2014 ESC guideline presented a novel risk prediction model (HCM Risk-SCD), which provided a calculated 5-year SCD risk (7, 8). Several studies illustrated that the HCM Risk-SCD model showed remarkable improvement in predicting the risk of SCD than previous models (9–11). However, the HCM Risk-SCD model resulted in lower sensitivity, incorrectly classifying more patients at SCD risk as low risk without ICD implantation (12, 13). The 2020 AHA/ACC guideline provided a new risk stratification and its external validations have not been reported (14).

The aims of this study were to (1) validate the SCD risk stratification recommended by the 2020 AHA/ACC guideline in Chinese HCM patients and (2) compare the ability of distinguishing high-risk SCD patients among SCD risk stratifications recommended by the 2011 ACCF/AHA guideline, the 2014 ESC guideline, and the 2020 AHA/ACC guideline.

## Methods

### Study Population

The retrospective observational study consisted of all consecutively evaluated patients who were diagnosed as HCM at the First Affiliated Hospital of Nanjing Medical University from January 1, 2013 to December 31, 2018. The diagnosis of HCM was based on the echocardiographic demonstration of an unexplained left ventricle (LV) hypertrophy (LV maximum wall thickness ≥15 mm) in the absence of any other cardiac or systemic disease capable of producing such a magnitude of hypertrophy (e.g., uncontrolled hypertension, valvular heart disease, or chemotherapy) (5, 7, 14). The exclusion criteria were (1) patients with HCM linked to Noonan's syndrome and Fabry's disease, glycogen storage disease, cardiac amyloidosis, and mitochondrial disease; (2) patients younger than 16 years old; (3) a history of an ICD for secondary prevention of SCD; (4) a history of surgical myectomy or alcohol septal ablation or heart transplant; or (5) patients who were lost to follow-up.

By viewing the patient's electronic medical record, we collected the baseline data, including demographics, comorbidities (atrial fibrillation, coronary heart disease, hypertension, cerebral infarction, and diabetes mellitus), and data of echocardiography and 24-h Holter monitoring and cardiac magnetic resonance (CMR). The study protocol was approved by the Clinical Studies and Ethics Committee of the First Affiliated Hospital of Nanjing Medical University.

### Follow-Up and Endpoints

The follow-up was conducted by clinical visits, reviews of medical records, and telephone interviews up to September 2020. The follow-up extended from the first evaluation to the endpoint or death from another cause. The endpoint was SCD event including SCD or an equivalent event (7, 15). SCD was defined as instant and unexpected death within 1 h of new symptoms in patients who were previously in a stable condition, or nocturnal death with no antecedent history of worsening symptoms (15). Appropriate ICD shock or successful resuscitation after cardiac arrest was considered equivalent to SCD (15). The appropriate ICD shock was in line with previous studies such as ICD interventions for ventricular fibrillation or fast ventricular tachycardia (>200 beats per minute) (7).

### SCD Risk Stratifications of Three Guidelines

Risk factors for SCD were assessed at baseline according to the following three guidelines, respectively. Missing data on risk factors were coded as absent.

#### 2011 ACCF/AHA Guideline

In the 2011 ACCF/AHA guideline (5), five major risk factors to estimate the risk for SCD were (1) family history of SCD, (2) unexplained syncope, (3) the presence of nonsustained ventricular tachycardia (NSVT) on Holter monitoring, (4) maximal left ventricular wall thickness (LVWT) ≥30 mm, and (5) abnormal blood pressure response to exercise (ABPRE). The NSVT and ABPRE were only considered major risk factors if at least one of the others was present. The patients with ≥1 major risk factor were supposed to be at high risk of SCD recommended for ICD implantation for primary prevention (Class IIa).

#### 2014 ESC Risk-SCD Model

The 2014 ESC guideline (7) presented a novel risk prediction model (HCM Risk-SCD), which provided a calculated 5-year SCD risk using seven risk factors: (1) age at time of evaluation; (2) family history of SCD; (3) maximal LVWT; (4) left atrial (LA) diameter; (5) maximal left ventricular outflow tract (LVOT) gradient; (6) documented NSVT; (7) unexplained syncope. According to the HCM Risk-SCD model, probability of SCD at 5 years = 1 – 0.998^exp (Prognostic Index)^, where Prognostic Index = 0.15939858 × maximal LVWT (mm) – 0.00294271 × maximal LVWT^2^ (mm^2^) + 0.0259082 × LA diameter (mm) + 0.00446131 × maximal LVOT gradient (mmHg) + 0.4583082 × family history of SCD + 0.82639195 × NSVT + 0.71650361 × unexplained syncope – 0.01799934 × age at evaluation (years).

Patients were divided into three groups according to the HCM Risk-SCD model: low risk <4%, intermediate risk 4% to <6%, and high risk ≥6%. A calculated 5-year SCD risk of ≥4% confers a recommendation (≥6%, class IIa; and between 4 and <6%, class IIb) for ICD implantation for primary prevention of SCD.

#### 2020 AHA/ACC Guideline

The 2020 AHA/ACC guideline provided a new risk stratification composed of four conventional (family history of SCD, unexplained syncope, NSVT, and maximal LVWT ≥30 mm) and three additional risk factors. The additional risk factors included LV systolic dysfunction (left ventricular ejection fraction, LVEF <50%), LV apical aneurysm, and extensive late gadolinium enhancement (LGE) by contrast-enhanced CMR imaging. The major risk factors include (1) family history of SCD, (2) unexplained syncope, (3) maximal LVWT ≥30 mm, (4) LV apical aneurysm, and (5) LV systolic dysfunction (LVEF <50%). NSVT and extensive LGE on CMR were considered as risk factors but not major risk factors. Similar to the 2011 ACCF/AHA guideline, patients at high risk of SCD recommended for ICD implantation should have at least one major risk factor (class IIa).

### Statistical Analysis

Continuous variables with a normal distribution are presented as mean ± SD and were compared using Student's *t*-test. Non-normally distributed continuous variables are presented as median with interquartile range and were compared using Mann–Whitney *U* test. Categorical variables are presented as frequencies (percentage) and were analyzed using the χ^2^ test or Fisher's exact test.

Receiver operating characteristic (ROC) curves were constructed to visualize the risk stratification performances, by plotting sensitivity against 1 – specificity with the area under the curve. The area under the curve of 0.5 indicates no predictive value and 1.0 indicates perfect discrimination. Kaplan–Meier survival curves showed survival rates free from the endpoint SCD, non-SCD death, and all-cause death among three risk stratifications using the log-rank test. To visually assess the efficiency of three risk stratifications to discriminate high-risk SCD patients who needed ICD implantation under the recommendation of guidelines, the number needed to treat (NNT) and its 95% CI were calculated based on Bender's method (16, 17). All statistical analyses were conducted using the IBM SPSS software version 25, and a two-tailed *p* value of less than 0.05 was considered statistically significant.

## Results

### Baseline Clinical Characteristics

The finial study population consisted of 511 patients with HCM. The baseline characteristics of these patients are presented in Table 1. Mean age was 59.8 ± 13.4 years, and 62.6% of patients were male. Baseline characteristics of patients with SCD were comparable with those of patients without SCD except for significantly lower LVEF (55.1 ± 15.2% vs. 63.9 ± 4.9%; *p* = 0.041), lower rate of coronary heart disease (0 vs. 21.8%; *p* = 0.049), higher rate of NYHA III/IV (33.3% vs. 11.9%; *p* = 0.029), multiform premature ventricular contractions (PVCs) (26.7% vs. 4.6%; *p* = 0.006) and NSVT (46.7% vs. 12.1%; *p* = 0.001), and higher rate of LVEF <50% (33.3% vs. 1.8%; *p* < 0.001) and apical aneurysm (13.3% vs. 0.2%; *p* = 0.002).

**Table 1:** Baseline clinical characteristics of the 511 HCM patients.

	**Patients without SCD event (*n* = 496)**	**Patients with SCD event (*n* = 15)**	**All (*n* = 511)**	***P*** **value**
Male	310 (62.5)	10 (66.7)	320 (62.6)	0.742
Age	59.7 ± 13.3	62.2 ± 14.3	59.8 ± 13.4	0.473
Symptoms				
Palpitation	205 (41.3)	7 (46.7)	212 (41.5)	0.679
Angina	153 (30.8)	3 (20.0)	156 (30.5)	0.570
Dyspnea	131 (26.4)	4 (26.7)	135 (26.4)	1.000
Syncope	37 (7.5)	2 (13.3)	39 (7.6)	0.320
Family history of HCM	17 (3.4)	1(6.7)	18(3.5)	0.420
NYHA III/IV	59 (11.9)	5 (33.3)	64 (12.5)	0.029
Atrial fibrillation	127 (25.6)	4 (26.7)	131 (25.6)	1.000
Coronary heart disease	108 (100)	0	108 (0)	0.049
Hypertension	294 (59.3)	8 (53.3)	302 (59.1)	0.645
Cerebral infarction	33 (6.7)	0	33 (6.5)	0.614
Diabetes mellitus	66 (13.3)	3 (20.0)	69 (13.5)	0.440
Multiform PVC	23 (4.6)	4 (26.7)	27 (5.3)	0.006
LAD	40.7 ± 6.2	42.5 ± 7.9	40.8 ± 6.3	0.294
LVDd	46.4 ± 4.4	51.4 ± 11.5	46.6 ± 4.8	0.117
LVEF	63.9 ± 4.9	55.1 ± 15.2	63.7 ± 5.6	0.041
Maximal LVWT	17.2 ± 3.3	18.1 ± 4.1	17.2 ± 3.3	0.262
LVOT gradient	6 (0,10)	5 (0,28)	6 (0,10)	0.770
LVOT obstruction	78 (15.7)	3(20)	81 (15.9)	0.717
Drug therapy				
Beta-blocker	412 (83.1)	13 (86.7)	425 (83.2)	1.000
Calcium channel blocker	30 (6.0)	2 (13.3)	32 (6.3)	0.240
ACEI/ARB	221 (44.6)	3 (20.0)	224 (43.8)	0.059
Amiodarone	40 (8.1)	3 (20.0)	43 (8.4)	0.124
ICD implantation	12 (2.3)	1 (6.7)	11 (2.2)	0.303
Risk factors of the 2020 AHA/ACC guideline				
Family history of SCD	4 (0.8)	0	4 (0.8)	1.000
Unexplained syncope	37 (7.5)	2 (13.3)	39 (7.6)	0.320
NSVT	60 (12.1)	7 (46.7)	67 (13.1)	0.001
Maximal LVWT ≥30 mm	2 (0.4)	0	2 (0.4)	1.000
LVEF <50%	9 (1.8)	5 (33.3)	14 (2.7)	<0.001
LV apical aneurysm	1 (0.2)	2 (13.3)	3 (0.6)	0.002
LGE	8 (1.6)	1 (6.7)	9 (1.8)	0.237
Number of risk factors in the 2020 AHA/ACC guideline				<0.001
0	443 (89.3)	7 (46.7)	450 (88.1)	
1	42 (8.5)	3 (20.0)	45 (8.8)	
≥2	11 (2.2)	5 (33.3)	16 (3.1)	
Number of risk factors in the 2011 ACCF/AHA guideline				0.663
0	453 (91.3)	13 (86.7)	466 (91.2)	
≥1	43 (8.7)	2 (13.3)	45 (8.8)	
2014 ESC Risk-SCD score (%/5 years)	1.8 ± 1.1	2.8 ± 2.2	1.8 ± 1.2	0.001
2014 ESC Risk-SCD Model				0.023
<4%	472 (95.2)	12 (80.0)	484 (94.7)	
4%-6%	19 (3.8)	2 (13.3)	21 (4.1)	
≥6%	5 (1.0)	1 (6.7)	6 (1.2)	

*Values are expressed as either mean ± SD, median (interquartile range), or number (percentage). HCM, hypertrophic cardiomyopathy; PVC, premature ventricular contraction; LAD, left atrial diameter; LVDd, left ventricular end-diastolic diameter; LVEF, left ventricular ejection fraction; LVWT, left ventricular wall thickness; LVOT, left ventricular outflow tract; ACEI/ARB, angiotensin-converting enzyme inhibitor/angiotensin receptor blocker; ICD, implantable cardioverter defibrillator; SCD, sudden cardiac death; NSVT, nonsustained ventricular tachycardia; LGE, late gadolinium enhancement*.

### SCD and ICD Implantation

During a mean follow-up of 4.7 ± 1.7 years, 15 (2.9%) patients (mean age 62.2 ± 14.3 years; 66.7% male; mean follow-up 3.1 ± 2.2 years) reached the endpoint. The 1-year and 5-year cumulative survival rates free from SCD events were 99.2 and 97.0%, respectively. The combined endpoint consisted of 14 SCD (mean age 62.0 ± 14.8 years; 64.3% female; mean follow-up 3.0 ± 2.2 years) and 1 appropriate ICD shock (age 65 years; female; follow-up 4.5 years). Out of 511 patients, 12 patients (2.3%) implanted ICD for primary prevention. Only one patient with ICD implantation suffered one appropriate ICD shock.

### Risk Groups of SCD Events

#### 2011 ACCF/AHA Guideline

**Figure 1:**
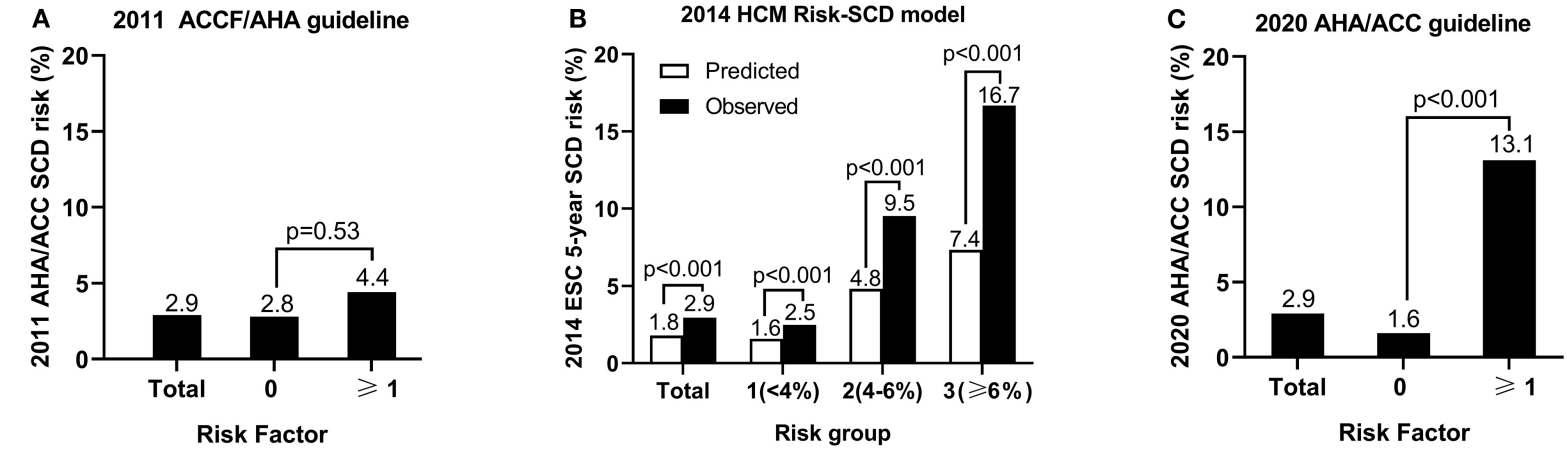
The sudden cardiac death (SCD) risk of groups based on the risk stratifications in the 2011 ACCF/AHA guideline **(A)**, the 2014 ESC guideline **(B)**, and the 2020 AHA/ACC guideline **(C)**.

On the basis of SCD risk stratification in the 2011 ACCF/AHA guideline, 45 patients (8.8%) had more than one risk factor and 466 (91.2%) had no risk factor. No statistically significant difference was demonstrated in the risk of SCD events between the patients with ≥1 risk factor and with no risk factor (4.4% vs. 2.8%, *p* = 0.53; Figure 1A).

#### 2014 ESC Risk-SCD Model

The mean calculated 5-year SCD risk of 511 patients was 1.8 ± 1.2% according to the 2014 ESC guideline. The observed SCD risk was 2.9% (15/511). The mean calculated 5-year SCD risk of low-, intermediate-, and high-risk group was 1.6 ± 0.8%, 4.8 ± 0.5%, and 7.4 ± 1.4%, while the observed incidence of SCD was 2.5, 9.5, and 16.7%, respectively. The predicted and observed risks per group are illustrated in Figure 1B. The observed risk of SCD events was significantly higher than the predicted risk per group (*p* < 0.001).

#### 2020 AHA/ACC Guideline

A total of 53 patients (10.7%) had more than one major risk factor based on the 2020 AHA/ACC guideline. The risk of SCD events significantly increased in patients with ≥1 risk factor compared with those with no risk factor (13.1% vs. 1.6%, *p* < 0.001; Figure 1C).

### Predictors of SCD Events

**Figure 2:**
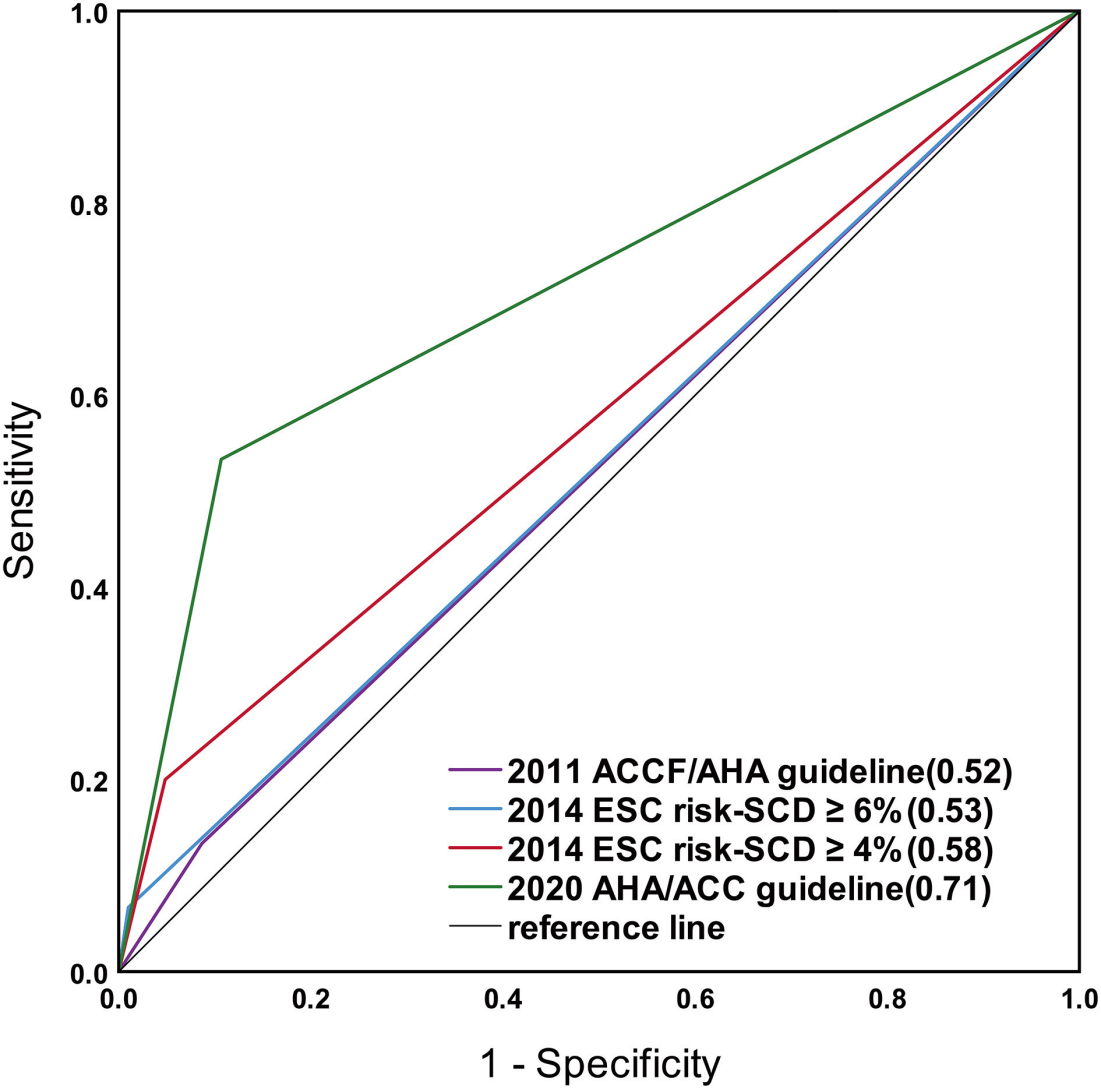
Receiver operating characteristic (ROC) curves for the risk prediction models of the 2011 ACCF/AHA, the 2020 AHA/ACC guidelines, and the 2014 ESC guideline with a cut-off level of 4 and 6%. The sudden cardiac death (SCD) risk stratification in the 2020 AHA/ACC guideline showed greater area under the curve (0.71) than the others.

Figure 2 shows ROC curves for the criteria of the 2011 ACCF/AHA, 2020 AHA/ACC guidelines, and the 2014 ESC guideline for a cut-off level of 4 and 6%.

#### 2011 ACCF/AHA Guideline

The area under the curve calculated for the study population using the 2011 ACCF/AHA guideline was 0.52 (95% CI 0.37–0.67, *p* = 0.76), discriminating between patients with and without SCD events, with a sensitivity and specificity of 13 and 91%.

#### 2014 ESC Risk-SCD Model

The area under the curve for the calculated risk was 0.68 (95% CI 0.54–0.81, *p* = 0.02) for the 2014 ESC model. For the 2014 ESC model with a cut-off level of 4%, the area under the curve was 0.58 (95% CI 0.41–0.74, *p* = 0.32), with a sensitivity and specificity of 20% and 95%. For the 2014 ESC model with a cut-off level of 6%, the area under the curve was 0.53 (95% CI 0.37–0.68, *p* = 0.71), with a sensitivity and specificity of 7 and 99%.

#### 2020 AHA/ACC Guideline

The area under the curve for the calculated risk using the 2020 ACC/AHA guideline was 0.71 (95% CI 0.56–0.87, *p* < 0.001), with a sensitivity and specificity of 53 and 89%.

**Figure 3:**
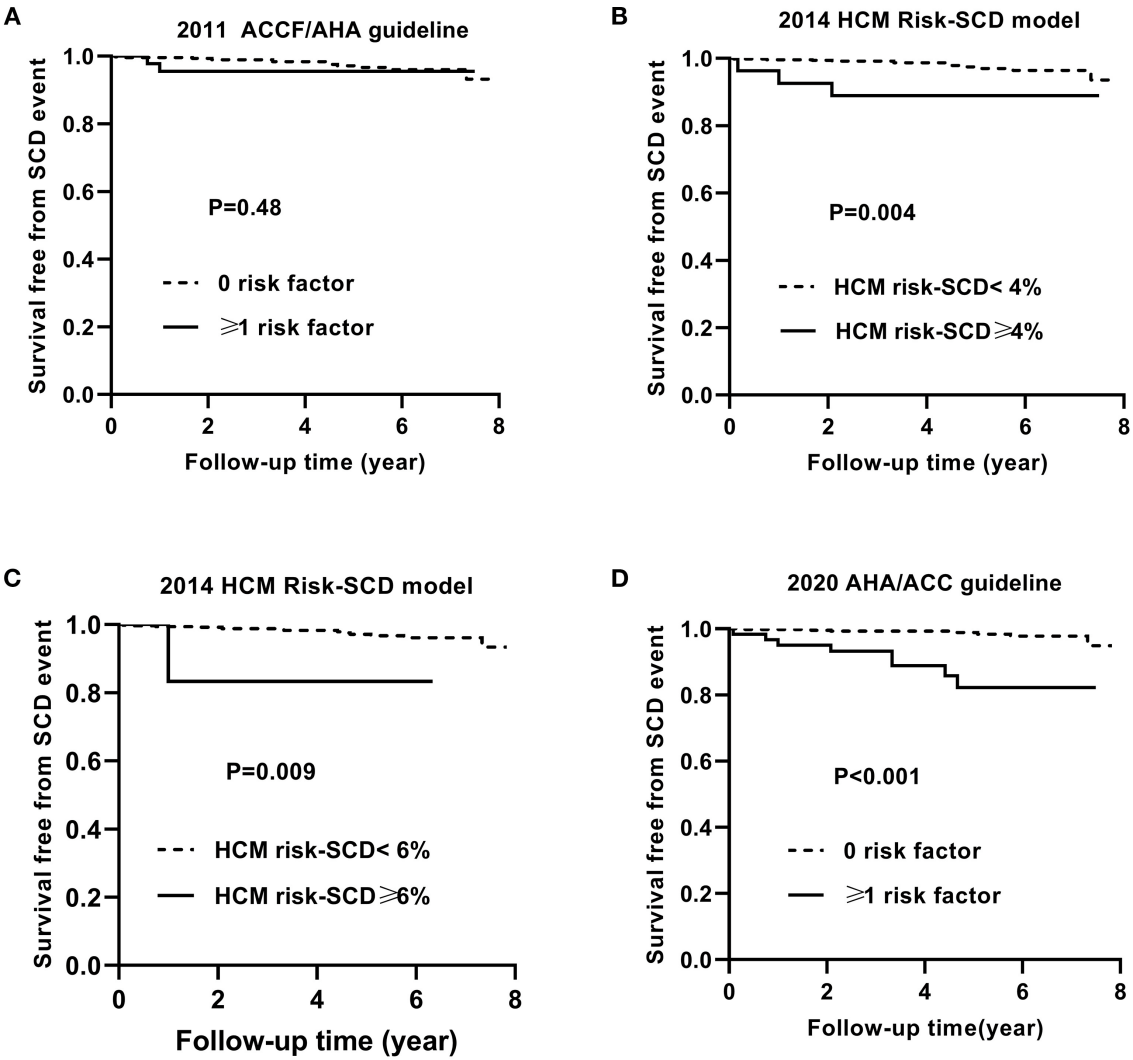
Kaplan–Meier curves for survival free from the sudden cardiac death (SCD) event by risk stratifications based on the 2011 ACCF/AHA **(A)**, the 2014 ESC guideline with a cut-off level of 4% **(B)** and 6% **(C)**, and the 2020 AHA/ACC guidelines **(D)**.

Figure 3 shows the Kaplan–Meier curves for survival free from the endpoint of SCD event based on the 2011 ACCF/AHA guideline (A), the 2014 ESC model with a cut-off level of 4% (B) and 6% (C), and the 2020 ACC/AHA guideline (D).

**Figure 4:**
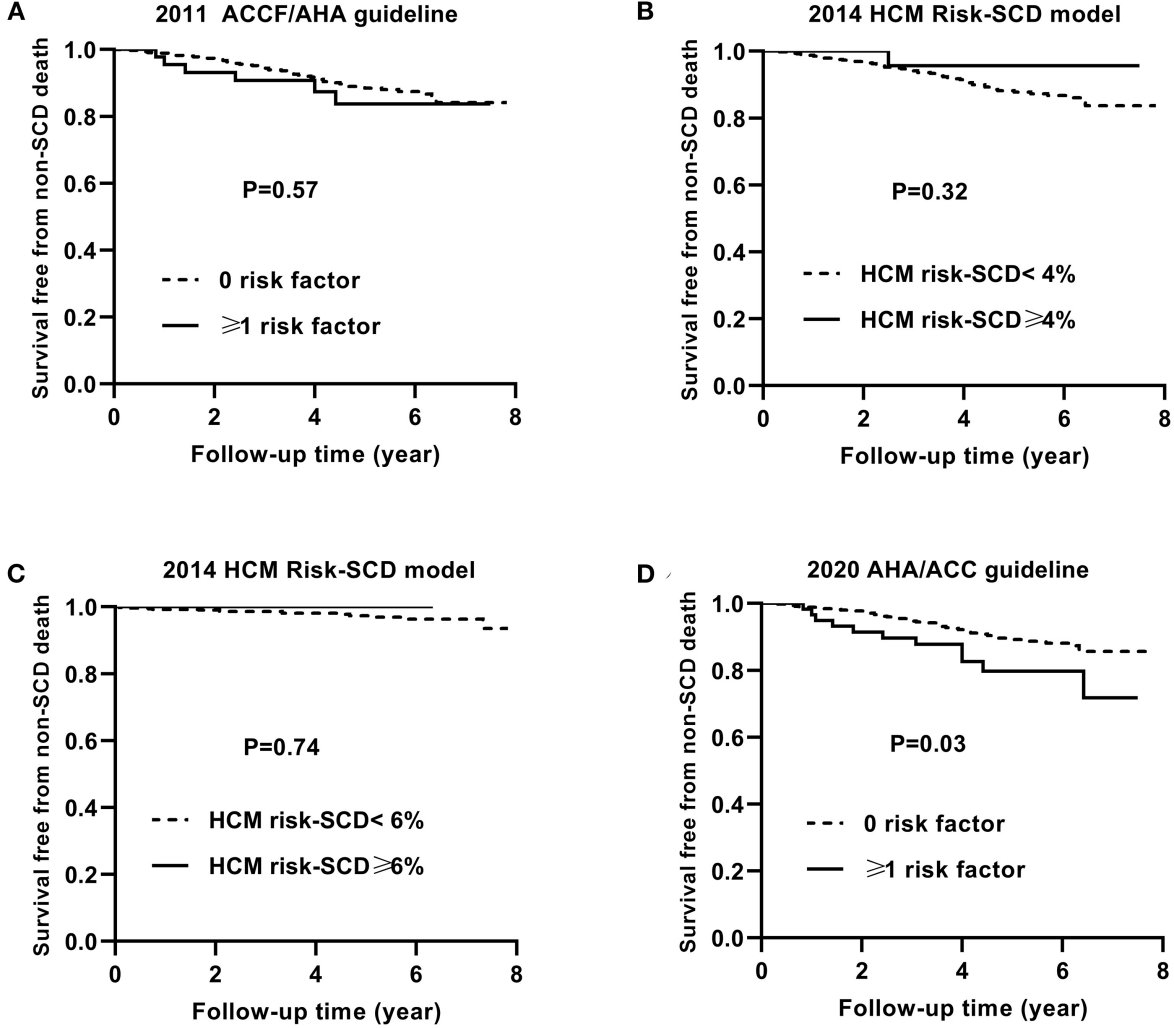
Kaplan–Meier curves for survival free from the non-SCD death by risk stratifications based on the 2011 ACCF/AHA **(A)**, the 2014 ESC guideline with a cut-off level of 4% **(B)** and 6% **(C)**, and the 2020 AHA/ACC guidelines **(D)**. SCD, sudden cardiac death.

**Figure 5:**
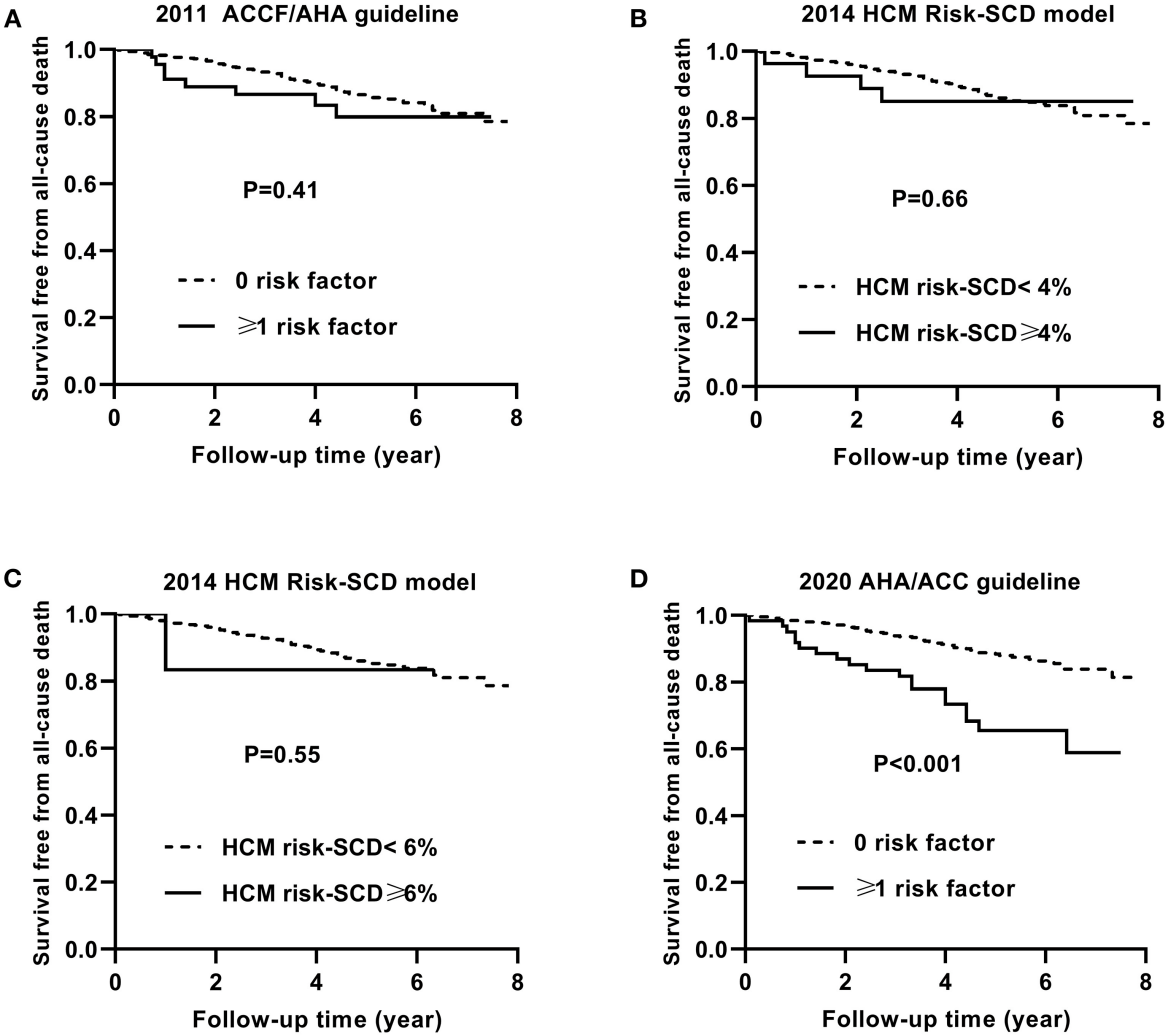
Kaplan–Meier curves for survival free from the all-cause death by risk stratifications based on the 2011 ACCF/AHA **(A)**, the 2014 ESC guideline with a cut-off level of 4% **(B)** and 6% **(C)**, and the 2020 AHA/ACC guidelines **(D)**.

Similarly, Figures 4, 5 show the Kaplan–Meier curves for survival free from the non-SCD death and all-cause death, respectively, based on three guidelines.

The non-SCD mortality and all-cause mortality in patients with ≥1 risk factor were higher than those in patients with 0 risk factor based on the 2020 ACC/AHA guideline (*p* = 0.03 and *p* < 0.001, respectively).

### Clinical Implications

To compare the predictive ability of SCD risk stratifications recommended by three guidelines, the odds ratio (OR) and number needed to treat (NNT) to prevent 1 case of SCD event are shown in Table 2. It shows that 8.7 ICD implantations are necessary to prevent 1 case of SCD in 5 years according to the 2020 ACC/AHA guideline. A total of 11.6 ICD implantations are necessary when using the 2014 ESC model with a cut-off level of 4%. However, it would be less effective to identify the patients at high risk of SCD with the criteria of the 2011 ACCF/AHA guideline or the 2014 ESC model with a cut-off level of 6%.

**Table 2:** Comparison of the odds ratio and number needed to treat for event predicting models.

	**High risk (ICD indicated)**	**Low risk(ICD not indicated)**	**OR (95% CI)**	***P*** **value**	**NNT (95% CI)**
2011 ACCF/AHA guideline	2/45	13/466	1.6 (0.4–6.6)	0.6	60.4 (7.3–∞)
2014 ESC risk-SCD ≥4%	3/27	12/484	4.9 (1.4–17.4)	0.04	11.6 (3.6–475.6)
2014 ESC risk-SCD ≥6%	1/6	14/505	7.0 (0.6–59.3)	0.2	7.2 (1.6–∞)
2020 AHA/ACC guideline	8/61	7/450	9.6 (3.3–25.1)	<0.001	8.7 (4.3–22.5)

*Values are expressed as either mean ± SD, median (interquartile range), or number (percentage). ICD, implantable cardioverter defibrillator; OR, odds ratio; NNT, number needed to treat*.

## Discussion

The study is the first comparative evaluation of SCD risk stratification methods recommended by the 2011 ACCF/AHA guideline, the 2014 ESC guideline, and the 2020 AHA/ACC guideline in a Chinese cohort with HCM. Also, it is the first independent validation of the SCD risk stratification method used in the 2020 AHA/ACC guideline. The most important finding of this study is that the SCD risk stratification used in the 2020 AHA/ACC guideline showed better discrimination than the other two risk stratifications in Chinese patients with HCM.

### Current Validations of Three Risk Stratifications

In the 2011 ACCF/AHA guideline, the identification of high-risk patients was based on five major binary risk factors. The risk of SCD increased with the number of major risk factors. Several studies demonstrated that the risk stratification in the 2011 ACCF/AHA guideline showed higher sensitivity and lower specificity, resulting in unnecessary ICD implantations (6, 18). The reported C-statistics ranged from 0.54 to 0.83 with the 2011 ACCF/AHA guideline in different cohorts (6, 9, 10, 18).

O'Mahony et al. provided a novel risk prediction model (HCM Risk-SCD) to stratify 5-year SCD risk, which was adopted as the 2014 ESC risk prediction model for primary prevention of SCD in HCM (8). It not only evaluated the SCD risk quantitatively with continuous variables rather than binary factors, but added into risk stratification new factors reflecting cardiac remodeling and disease progression (such as maximal LVWT, LA diameter, and LVOT gradient). O'Mahony et al. further conducted an international multicenter cohort study including more than 3,700 patients with HCM to verify the significant prediction of HCM Risk-SCD model (11). The reported C-statistics varied from 0.69 to 0.93 with the 2014 ESC guideline (9, 10, 18–20). Besides, studies from Asian HCM population demonstrated that the HCM Risk-SCD model resulted in lower sensitivity, missing more patients at SCD risk who do not receive ICDs (12, 19, 21).

The 2020 AHA/ACC guideline provided a new risk stratification composed of four conventional and three additional risk factors (LV systolic dysfunction, LV apical aneurysm, and extensive LGE by contrast-enhanced CMR imaging). The new factors included in the 2020 AHA/ACC guideline were mainly derived from high-risk patients with HCM in Western population (14). However, whether the SCD risk stratification method in the 2020 AHA/ACC guideline can be applied to Chinese patients with HCM is still questionable. Our study was to validate and compare the predictive value for SCD in Chinese HCM patients.

### New SCD Risk Factors of the 2020 AHA/ACC Guideline

The ROC curves vividly suggested that the 2020 AHA/ACC guideline strategy had better discrimination performance than the other two methods in our cohort. This may be attributed to the new major risk factors in the 2020 AHA/ACC guideline strategy (14): LV systolic dysfunction (LVEF <50%) and LV apical aneurysm. In a retrospective study published by Harris et al. in 2006 (22), end-stage HCM (LVEF <50%) were identified in 44 (3.5%) of 1,259 study patients. Also, investigators found that LV systolic dysfunction was associated with increased mortality rate (11% per year) and regarded as a sudden death risk factor. Maron et al. conducted a study in 118 (4.8%) end-stage HCM patients from 2004 to 2017 at the Tufts HCM Institute (23). The follow-up was up to December 31, 2018, and the study suggested that SCD events were fivefold more frequent in end-stage HCM patients than in patients with preserved LV systolic function (2.4 vs. 0.5%/year; *p* = 0.006). In our study, 14 (2.7%) were identified with LV systolic dysfunction in 511 HCM patients. Of 14 patients with LV systolic dysfunction, 4 (28.6%) suffered SCD and all of them did not implant ICD. Primary prevention ICDs should be considered in HCM patients with LV systolic dysfunction. In aspect of LV apical aneurysm, Igarashi et al. conducted an electrophysiological study in 15 patients with HCM and LV apical aneurysm (24). It showed that endocardial radiofrequency catheter ablation of LV apical aneurysm effectively suppressed monomorphic VT which decreased the mortality rate related to SCD. In a cohort of 93 patients with HCM and apical aneurysms, Rowin and colleagues demonstrated that LV apical aneurysm could be considered as a risk factor of SCD (25). It has been reported that LV apical aneurysms are present in up to 2% of patients with HCM (26). In the present study, 3 (0.6%) of 511 patients were identified with LV apical aneurysm and two patients reached the endpoint of SCD events.

In addition, the 2020 AHA/ACC guideline also includes the risk factor extensive LGE by CMR imaging, but it is not a major risk factor (14). CMR imaging with LGE can be used to detect and quantify myocardial fibrosis and scarring (27), which was associated with increased risk for future potentially lethal ventricular arrhythmias (28–31). Although several studies have promoted a threshold for extensive LGE of ≥15% of the LV mass as representing a significant increase in SCD risk (30, 31), no consensus is achieved on the methods used to quantify LGE and further verification is still needed (14). HCM is mostly caused by mutations in various genes encoding proteins of the cardiac sarcomere (32). Girolami et al. reported that patients with double or triple mutations are at increased risk of end-stage progression and ventricular arrhythmias (33). But whether genetic testing needs to be included in the risk stratification of SCD is uncertain because of current limited data (34–36).

### Lower Sensitivity of Three Risk Stratifications

The accurate risk stratification can not only identify the patients at a high risk of SCD to implant ICDs for primary prevention, but avoid overtreatment in low-risk patients. All three SCD risk stratifications in the present study showed lower sensitivity, resulting in significant proportion of patients experiencing SCD events that were misclassified into the low-risk patients. Thirteen (2.8%) patients experiencing SCD events were misclassified as low-risk patients by the 2011 ACCF/AHA guideline, twelve (2.3%) by the 2014 ESC model, and seven (1.6%) by the 2020 AHA/ACC guideline. The reasons of lower sensitivity can be listed as here. First, in the 2011 ACCF/AHA guideline, the risk factor ABPRE was not available in our cohort because we did not routinely assess the ABPRE in clinical practice. This may lead to lower sensitivity in our cohort than the previous reported sensitivity in cohorts from Western population (6, 18). Second, the reported sensitivity and specificity of the 2014 ESC model varied in different studies (8, 9, 12). The sensitivity in our study were similar to the one in a study composed of 1,629 HCM patients published by Maron et al. (a much lower sensitivity of 9%, but higher specificity of 96%) (12). The study indicated that the area under the curve of 2014 ESC model appeared to be higher because of its greater specificity. However, this may be at the expense of reduced sensitivity and thus missed appropriate ICD therapy. The actual incidence of 5-year SCD was higher than the predicted risk in per group in this study, reflecting that the model underestimated the risk of SCD and was not applicable to the Chinese HCM population. Third, although all three SCD risk stratifications showed low sensitivity, the sensitivity of the one recommended by the 2020 AHA/ACC guideline was highest.

The factor LV apical aneurysm in the 2020 AHA/ACC guideline may have been under-diagnosed in our study, as only 20 (4.3%) patients were evaluated by CMR imaging. Compared with CMR imaging, echocardiography can underestimate the detection rate of LV apical aneurysm (25, 37). It needs further researches to validate the risk stratification in HCM patients undergoing CMR. More attention should be paid to seeking for a SCD risk stratification applied to the Chinese HCM population with both higher sensitivity and specificity.

### ICD Implantation for Primary Prevention of SCD

Several studies use NNT to further evaluate the specificity of SCD risk stratification recommended by guidelines (10, 38, 39). A smaller NNT represents a higher specificity, indicating that a smaller amount of ICD needs to be implanted to prevent 1 SCD. The current guidelines recommend ICD implantation for high-risk SCD HCM patients. Patients at high risk of SCD can be protected by ICD implantation for primary prevention of SCD, but this protection comes at a price of inappropriate shocks and device-related complications (40). So it is necessary to seek for the real high-risk patients who are eligible for ICD implantation by an accurate risk stratification. According to the 2020 ACC/AHA guideline, only 8.7 ICD implantations are necessary to prevent 1 case of SCD in 5 years. On the precondition that high-risk patients are protected by ICD implantation, reducing unnecessary ICD implantation is beneficial to reduce the incidence of its potential complications and the economic burden of patients.

Current guidelines all recommend a shared-decision making process that takes into consideration the SCD risk stratification of the patient as well as physician judgment and patient preference (5, 7, 14, 41). In clinical practice, it is noteworthy that whether to implant an ICD does not only depend on risk stratification, but also related to physician judgment (the effect of using antiarrhythmic drugs) and patient preference (the will and economic conditions of patients). ICD implantation might also be limited by the will or economic conditions of patients because of its high cost and invasiveness. These factors cannot be accurately assessed in a retrospective study resulting in a relatively significant bias.

## Limitations

The limitations of our study should be acknowledged. First, this study was conducted in a single tertiary center with potential selection bias, so the patient population might not represent the general population with HCM. Further larger, multicenter, and prospective studies are warranted to confirm our results. Second, the comparison between different risk models is limited because of the small number of SCD events, and it is less effective to identify which one is an independent risk factor of SCD in our study. Third, the risk factors recommended by the 2011 ACCF/AHA guideline and 2020 AHA/ACC guideline included in our study were incomplete, leading to a relatively conservative result. The risk factor ABPRE in the 2011 ACCF/AHA guideline was not assessed in our cohort. Also, it was less accurate to evaluate the new major risk factor LV apical aneurysm in the 2020 AHA/ACC guideline with transthoracic echocardiography. These led to an underestimated result of risk stratifications. However, these data presented the validation of different SCD risk stratifications in Chinese HCM patients from real world.

## Conclusion

In the present study, the SCD risk stratifications recommended by the 2011 ACCF/AHA guideline, the 2014 ESC guideline, and the 2020 AHA/ACC guideline all showed lower sensitivity, resulting in a significant proportion of patients experiencing SCD events that were misclassified into the low-risk patients. However, the SCD risk stratification recommended by the 2020 AHA/ACC guideline showed a better discrimination than previous stratifications in Chinese patients with HCM.

## Data Availability Statement

The raw data supporting the conclusions of this article will be made available by the authors, without undue reservation.

## Ethics Statement

The study protocol was approved by the Clinical Studies and Ethics Committee of the First Affiliated Hospital of Nanjing Medical University.

## Author Contributions

YD contributed to the conception and design of the work. YD, WY, CC, JJ, and WZ contributed to the acquisition and interpretation of data for the work. WY and WZ contributed to the analysis of data for the work. YD drafted the article. FZ, BY, XL, and XZ critically revised the article. All authors contributed to the article and approved the submitted version.

## Conflict of Interest

The authors declare that the research was conducted in the absence of any commercial or financial relationships that could be construed as a potential conflict of interest.

## Publisher's Note

All claims expressed in this article are solely those of the authors and do not necessarily represent those of their affiliated organizations, or those of the publisher, the editors and the reviewers. Any product that may be evaluated in this article, or claim that may be made by its manufacturer, is not guaranteed or endorsed by the publisher.
